# Prediction of soil shear strength using hybrid machine learning approaches for performance and interpretability analysis

**DOI:** 10.1038/s41598-026-57764-z

**Published:** 2026-06-12

**Authors:** Muhammad Suliman, Maaz Khan, Touqeer Ali Rind, Farhan Ullah, Sabila Khan, Zafar Mahmood

**Affiliations:** 1 Department of Civil and Environmental Engineering, Nagaoka University of Technology, Nagaoka, Niigata Japan; 2 Department of Civil Engineering, Ghulam Ishaq Khan Institute of Engineering Sciences and Technology, Topi, 23640 Pakistan; 3 Department of Civil Engineering, University of Engineering and Technology Peshawar, Jalozai Campus, Pabbi, Pakistan; 4 Civil Engineering Department, College of Engineering, Imam Mohammad Ibn Saud Islamic University (IMSIU), Riyadh, Saudi Arabia; 5 Department of Civil Engineering, Mehran University of Engineering and Technology, SZAB Campus Khairpur Mir’s, Sindh, 66020, Pakistan, Khairpur Mir’s, Pakistan

**Keywords:** Soil shear strength, Random forest (RF), Multi-expression programming (MEP), SHAP analysis, Geotechnical data modeling, Engineering, Environmental sciences, Mathematics and computing, Natural hazards

## Abstract

Shear strength of soil plays an essential role in geotechnical properties affecting construction stability. Conventional laboratory testing for determining soil shear strength is often expensive and time-consuming. Therefore, machine learning (ML) methods were employed to predict soil shear strength using geotechnical parameters compiled from previously published literature based on investigations conducted at the Le Trong Tan Geleximco project in western Hanoi, Vietnam. In total, there were 202 samples analyzed, including parameters such as depth, sand percent, loam percent, clay percent, moisture content, wet density, dry density, void ratio, liquid limit, plastic limit, plasticity index, and liquidity index. Four predictive models have been developed and analyzed, including Multiple Linear Regression (MLR), Support Vector Machine (SVM), Random Forest (RF), and Multi-Expression Programming (MEP). The model performance was evaluated using several parameters like coefficient of determination (R^2^), Root Mean Square Error (RMSE), Mean Absolute Error (MAE), residual analysis, and Taylor diagrams. RF showed the best predictive performance, with training R^2^ = 0.9527 and testing R^2^ = 0.8578, along with the lowest prediction error. The SVM model performed impressively in terms of prediction, while the MEP model demonstrated satisfactory accuracy along with the added benefit of mathematical equation formulation. Conversely, the MLR model was relatively less accurate because of its inability to deal with nonlinear relations. Moreover, the SHAP analysis revealed that liquid index, moisture content, and plasticity index were the most critical factors influencing the prediction of soil shear strength. This research has established that modern machine learning models like RF and SVM prove to be efficient at modeling the nonlinear nature of soil characteristics.

## Introduction

Soil shear strength is a basic geotechnical property that is the basis for the stability of all civil engineering structures^1^. It determines the carrying capacity of foundations and the stability of earth structures (retaining structures, slopes, and embankments)^2^. In classical soil mechanics, shear strength is characterized by two main parameters: cohesion (c) and the internal friction angle (φ)^3^. Such parameters are usually identified through laboratory tests (e.g., triaxial compression test or direct shear test) or estimated through field tests (SPT and CPT). However, conventional laboratory testing to obtain c and φ is time-consuming, costly, and requires high levels of expertise^4^ In addition to the fundamental c and φ values, there are numerous other factors that affect the shear strength of the soil, including the moisture content, plasticity (liquid and plastic limits), void ratio, specific gravity, and stress history^5^.

Over the past years, sophisticated ML algorithms have been appealing to geotechnical issues due to the capability to simulate complex, nonlinear associations among several soil parameters^6^ . These empirical methods can train behavior using vast libraries of soil data and may be more generalizable than traditional regression-based correlations. MLR, SVM, RF and MEP are among other methods of ML that have been used to predict important soil properties with great success^7,8^. MLR is a statistical technique that models linear relationships between a dependent variable and multiple independent variables, offering simplicity and interpretability^9^. In contrast, SVM is a kernel-based algorithm that identifies an optimal hyperplane to capture complex non-linear relationships, delivering robust performance even with limited data. Similarly, RF is an ensemble-based algorithm that improves prediction accuracy and reduces overfitting by combining multiple decision trees^10^. The MEP is an evolutionary symbolic regression algorithm that generates explicit mathematical equations, providing valuable insight into the underlying physical relationships^11,12^.

RF-based hybrid models have demonstrated a high degree of precision in the estimation of undrained shear value of soils, RF with SHAP analysis has been employed to identify the strength of geopolymer-stabilized clayey soils^13,14^. In the same way MEP has been applied to determine the unconfined compressive strength of the fly-ash-treated alkali-contaminated soil and explicit mathematical equations are obtained to assess the interaction between complex parameters^15^. Moreover, MEP and GEP comparison of the expansive soils in predicting compaction behavior revealed that MEP is practical in the modelling of nonlinear geotechnical behavior^16^. The unconfined compressive strength of diverse soil types has been predicted using MLR with physical, grain-size, and binder composition parameters, achieving notable accuracy and offering transparent regression coefficients for practical interpretation^17,18^. Likewise, SVM has been employed to estimate the shear strength of soils from clay content, moisture content, void ratio, and Atterberg limits, producing strong correlations (R = 0.9–0.95) and substantially outperforming conventional MLR approaches in capturing non-linear soil behavior^19^.

Even though machine learning has enhanced geotechnical modeling, there has not been much research on the comparisons of the predictive ability and interpretation of various models used in the determination of soil shear strength. Furthermore, it is commonly alleged that ML models are black boxes, thus, tools like SHAP (Shapley Additive Explanations) are becoming common to interpret their results. RF and neural network models combined with SHAP analysis have been developed for predicting undrained shear strength, where SHAP analysis identified pre-consolidation stress as the dominant predictor.

In the same vein, the contemporary methods of empirical data are ineffective in considering the nonlinear interactions between soil parameters. Thus, the aim of this work is to develop and compare four different ML models: MLR, SVM, RF, and MEP, to predict soil shear strength based on the available data. as well as to apply explainable AI methods to determine the most significant factors and analyze the correlation between the prediction accuracy and model interpretability.

## Methodology

**Fig. 1:**
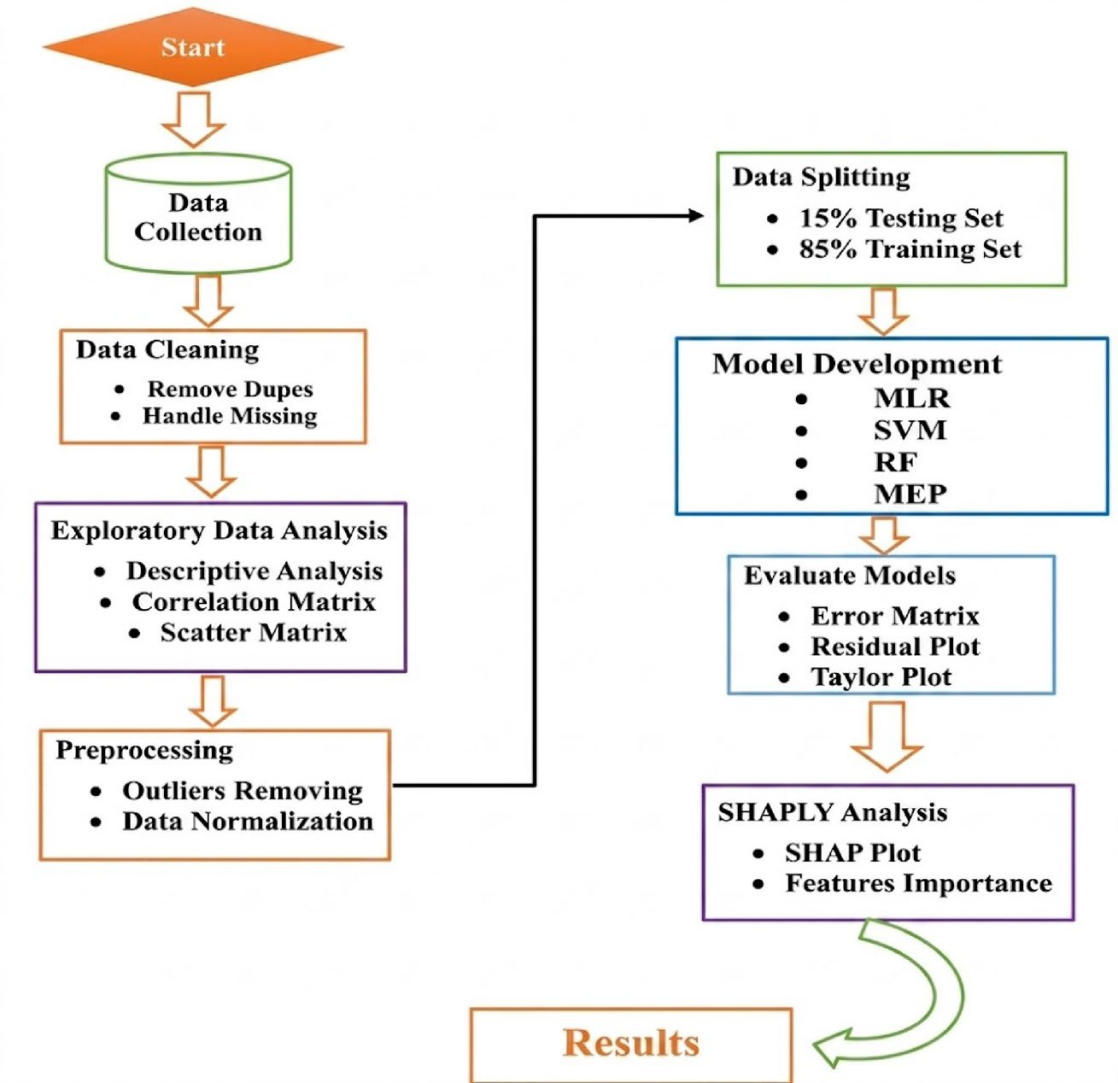
Methodology flow chart.

The methodology approach to be adopted in the study is a systematic machine learning pipeline which is illustrated in Fig. 1. Data collection was then followed by Data Cleaning which was stringent to filter out the duplicates and deal with the missing values and ensure data integrity. Then, an Exploration Data Analysis (EDA) has been conducted with care to Descriptive Analysis, Correlation Matrix, and Scatter Matrix visualization to get a sense of the underlying distribution and relationships of the data. The data went into the Preprocessing stage where outliers were eliminated and then the data normalization was performed. To train the model, the data was divided into 85% Training Set and 15% Testing Set. The essence of the research consisted in the ML Model Development, with the application of four advanced ML algorithms; MLR, SVM, RF, and MEP models. The Evaluate Models stage was used to measure model performance, and some of the important metrics that were used to measure model performance are the Error Matrix, Residual Plot, and Taylor Plot. Lastly, interpretability of the models was done through SHAP Analysis that created SHAP Plots and calculated Feature Importance, and this would give a clear picture of the predictive mechanisms of the models Developed.

### Data collection and cleaning

The initial dataset consists of 249 data points extracted from previously published geotechnical investigations conducted for the Le Trong Tan Geleximco Project, located in western Hanoi, Vietnam^20^. According to the original study, the site investigation activities were carried out in April 2009, and the subsurface profile primarily comprised soft clayey and silty soils, which are common in the Hanoi region. The original investigation employed borehole-based soil sampling techniques to evaluate the ground conditions^20^. Pre-processing of data is an important stage of the machine learning pipeline since the quality of the input data is the sole determinant of the model performance, convergence, and its capacity to extrapolate to novel^21^. So, a data pre-processing process was thereafter undertaken with the use of MATLAB data cleaner app. It entailed the extraction and removal of duplicates, treatment of missing data, and the dispensing of outliers in a stratified manner. Such strict cleaning is critical to the integrity and quality of the features which form the basics of training strong predictive models and avoid the propagation of errors or biases^22^. After all these measures, the ultimate dataset was narrowed down to 202 data points, on which model training and validation were performed.

### Descriptive analysis

Table 1 presents the descriptive statistics of the soil parameters, including measures of central tendency and variability such as mean, standard deviation, variance, skewness, and kurtosis. The findings show that the mean value of the loam percentage is highest at 50.57% followed by moisture content 36.14% and clay percentage 28.01%, which means that the soil is mostly loamy with the medium clay content. The sample depth ranges between 1.20 m and 19.80 m and mean depth of the sample is 7.23 m, indicating that the samples have been collected at shallow and moderately deep levels. The mean wet and dry densities are 1.82 g/cm^3^ and 1.34 g/cm^3^, respectively, which represents normal behavior of compacted soil. Further, the liquid limit and plastic limit have a larger mean value 42.31% and 27.67% and the shear strength is comparatively low 0.34 (kg/cm^2^) which means moderate plasticity and low shear resistance.

**Table 1:** Descriptive Analysis.

	Mean	Standard Deviation	Variance	Skewness	Kurtosis	Minimum	Median	Maximum
Depth of the Sample (m)	7.23	4.48	20.08	0.51	− 0.81	1.20	6.50	19.80
Sand Percentage (%)	21.04	7.95	63.17	0.37	− 0.54	6.79	21.40	42.60
loam Percentage (%)	50.57	7.65	58.54	− 0.54	− 0.13	27.70	51.30	63.70
clay percentage (%)	28.01	1.61	2.58	− 0.81	0.20	23.30	28.30	31.60
Moisture content (%)	36.14	7.04	49.61	0.11	− 1.12	20.60	36.20	49.80
wet density(g/cm3)	1.82	0.08	0.01	0.06	− 1.37	1.70	1.82	1.97
dry density (g/cm3)	1.34	0.12	0.02	0.09	− 1.25	1.14	1.33	1.62
void ratio	1.03	0.18	0.03	0.11	− 1.26	0.67	1.03	1.37
liquid limit (%)	42.31	5.83	33.98	0.33	− 0.96	32.10	41.30	54.10
Plastic limit (%)	27.67	5.37	28.79	0.31	− 0.93	16.50	27.00	39.60
plasticity index	14.65	1.72	2.97	− 0.82	− 0.10	10.00	15.00	17.50
liquidity index	0.58	0.15	0.02	− 0.45	− 0.60	0.25	0.60	0.88
Shear Strength (kg/cm^2^)	0.34	0.09	0.01	0.60	− 0.68	0.18	0.32	0.55

**Fig. 2:**
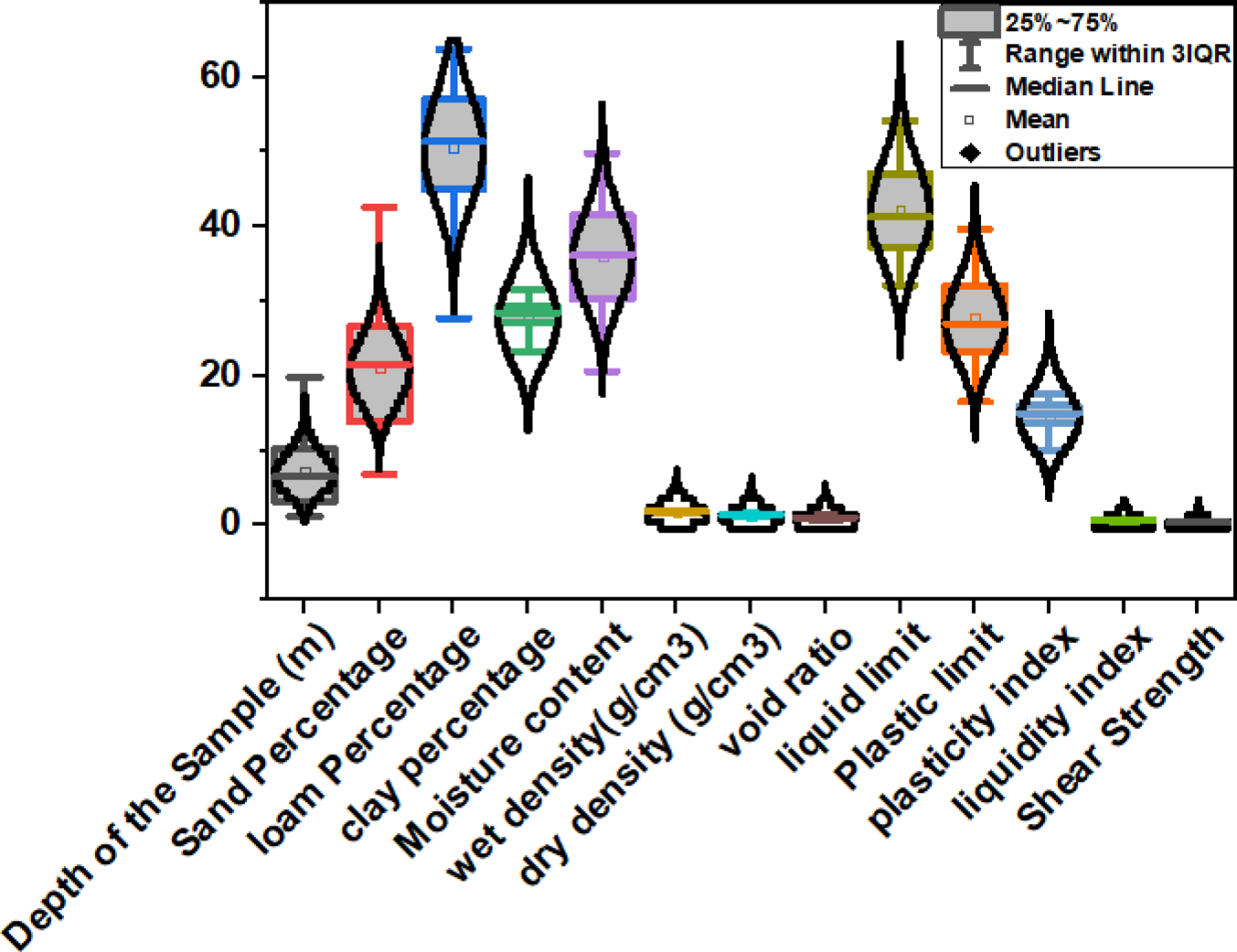
Data distribution plot.

These results are graphically complemented by Fig. 2 that demonstrates violin plots of each parameter showing the range, variability, and distribution. A wide spreading of sand, loam, and moisture contents are also present in the plot, and the variability is high, whereas the values of such properties as dry density and shear strength are not widely distributed but are more consistent, which means they have fewer deviation ranges.

### Correlation analysis

**Fig. 3:**
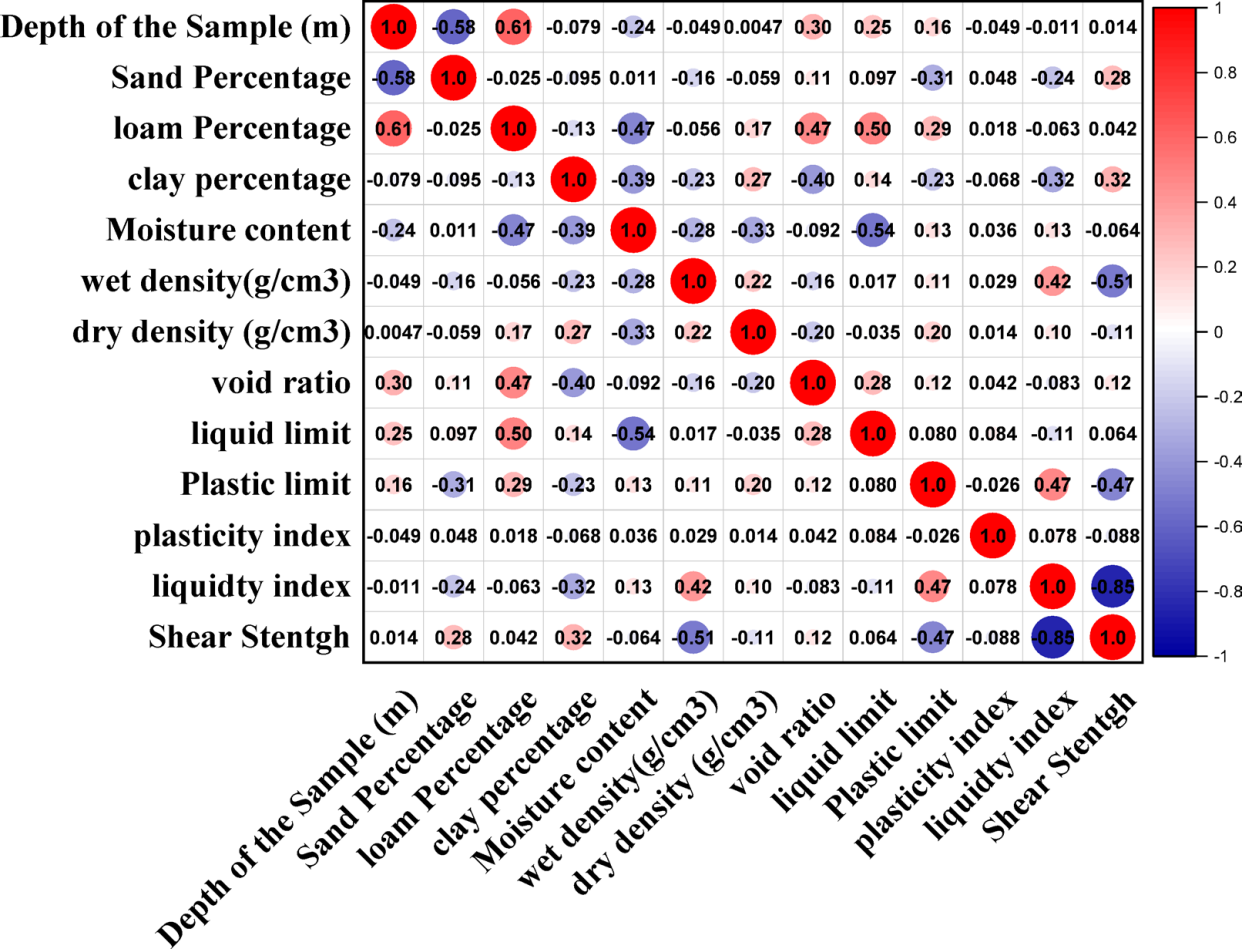
Correlation Matrix.

Examining the linear and non-linear correlations between features and outputs is crucial for understanding the interplay within the dataset^23^. The correlation analysis conducted with geotechnical variables displayed in Fig. 3 indicated that there are several critical inter-dependences between the soil parameters. The correlation matrix analysis has revealed several significant relationships between the tested parameters of the soil, primarily relating to shear strength, a key property of the material. Shear strength is found to have a strong negative correlation with liquidity index (− 0.85) and wet density (− 0.51), implying that a high liquidity index and a high wet density have a significant impact on the structural integrity of the material. At the same time, moderate positive correlations are found between shear strength and clay content (0.32) and sand content (0.28). In terms of stratigraphy, sample depth is found to have a strong positive correlation with loam content (0.61), along with a corresponding negative correlation with sand content (− 0.58), implying a significant change in composition at a greater depth. Moisture content is found to have an inverse relationship with liquid limit (− 0.54) and loam content (− 0.47), suggesting a complex relationship between composition and Atterberg limits for the tested sample of soil.

## Model development

MLR, SVM, RF, and MEP algorithms were developed to estimate the predictive performance of soil shear strength focusing on the combination of explainability and high predictive accuracy. The necessary geotechnical is based on the laboratory and field test data. A preprocessing of all data was carried out before modeling to increase consistency and reliability. Missing values were filled in using median imputation and Min–Max was used to put all features on the same scale. The raw dataset was preprocessed and separated into 85% training and 15% test to gain as much learning as possible and adequate test to generate a generalization. Key statistical indices, Coefficient of Determination (R^2^), Root Mean Square Error (RMSE), and Mean Absolute Error (MAE) were used to evaluate the model performance.

### Multilinear regression (MLR) model

MLR model has been developed to derive the correlation between shear strength (y, kg/cm^2^) and the geotechnical inputs. It is assumed that there is an additive linear relationship between the dependent variable y and the independent variable set, meaning that the dependent variable can be defined as the sum of weights and independent variables. Before proceeding with the modeling process, all variables were preprocessed to avoid any missing data. Moreover, the data set was split into two subsets; one was used 85% for training purposes, and the other was used 15% for testing to evaluate the MLR’s ability to generalize the unseen samples. The MLR coefficients have been computed using the least squares method, which minimizes the sum of squared residuals between observed and predicted data. This model serves as a baseline to measure improvements that can be achieved by MEP. MLR performance is measured in terms of R^2^, RMSE, and MAE statistics, indicating the model’s inability to capture complex nonlinear phenomena in soil mechanics.

### Support vector machine (SVM) model

In this study, the SVM regression model was optimized by selecting the RBF kernel, which is appropriate for modeling the relationship that exists between the soil properties and the shear strength of the soil since it can handle non-linear relationships. The optimized SVM regression model achieved the optimum result after setting the penalty parameter (C), kernel coefficient (γ), and insensitive loss parameter to 100, 0.1, and 0.05, respectively. The optimization process involved hyperparameter tuning using cross-validation techniques to minimize overfitting and maximize performance.

### Multi expression programming (MEP) model

Multi Expression Programming algorithm is an advanced genetic programming, which represents and simultaneously develops multiple candidate expressions in a specific chromosome, to find the best mathematical relationship between input and output. This method works especially well in geotechnical prediction exercises as it offers clear-cut symbolic equations, which help to uncover lying behind the scenes physical relationships among the soil properties and shear strength^24^. Optimization of MEPs in this study was done by experimentation. The optimum configuration was one with a population of 100, 500 generations, a chromosome length of 40 genes, crossover rate of 0.8 and a mutation rate of 0.05. The functionality that got utilized was +, −, multi n, division, power, square root, and this made it adaptable to produce sophisticated nonlinear relationships. The advantage of the MEP is that it provides human readable and transparent equations, rather than black-box predictions, which is suitable in the engineering decision support.

### Random forest (RF) model

One machine learning model used for forecasting and categorization is the Random Forest. A significant volume of high-quality data is essential for efficient data collection to train machine learning algorithms and artificial intelligence models^25^. RF effectively balances bias and variance and performs well on large and high-dimensional datasets. RF is versatile model that can be effectively used for both classification and regression problems. They are less prone to overfitting during the training phase due to the inherent randomness in their structure. While computational requirements may increase as the number of features grows, Random Forests remain valuable for applications requiring low model complexity, such as certain medical diagnostics, financial modeling, or environmental science problems. Random Forest (RF) regression model was created by means of an ensemble of the decision trees trained on bootstrap samples of the data. There were to be 300 trees, and the 8 depth was optimized to ensure that overfitting did not occur. A random selection of a subset of features at each node, which was the square root of the overall number of input variables, was used. The minimum number of samples that were required to split an internal node and to create a leaf node was set by 2 and 1, respectively. The splitting criterion was the mean squared error, and a constant random seed was used to ensure reproducibility.

### Performance assessment

It is crucial to assess the estimation capacity of machine learning models using a range of statistical measures to guarantee the development of robust models that may forecast the output with low error-free outcomes. This work uses three of the most widely used error measures to assess the models’ performance throughout both the training and testing phases. The error measures used, their mathematical formulations, their applicability, and the ideal values at which models should be accepted are listed in Table 2.

**Table 2:** Error metrix summary.

No	Metric	Abbreviation	Formula	Criteria
1	Root Means Square Error	RMSE	$RMSE = \sqrt {\frac{1}{n}\mathop \sum \limits_{{i = 1}}^{n} \left( {y_{i} - \hat{y}_{i} } \right)^{2} }$	Near to zero
2	Coefficient of Determination	R^2^	${R}^{2}=1-\frac{\sum_{i=1}^{n}{\left({y}_{i}-\hat{y}\right)}^{2}}{\sum_{i=1}^{n}{\left({y}_{i}-\overline{y}\right)}^{2}}$	R^2^ > 0.8
3	Mean Absolute Error	MAE	$\mathrm{M}\mathrm{A}\mathrm{E}=\frac{1}{n}\sum_{i=1}^{n}\left|{y}_{i}-{\hat{y}}_{i}\right|$	Near to zero

## Results and discussion

### Regression analysis

#### Multilinear regression analysis

**Fig. 4:**
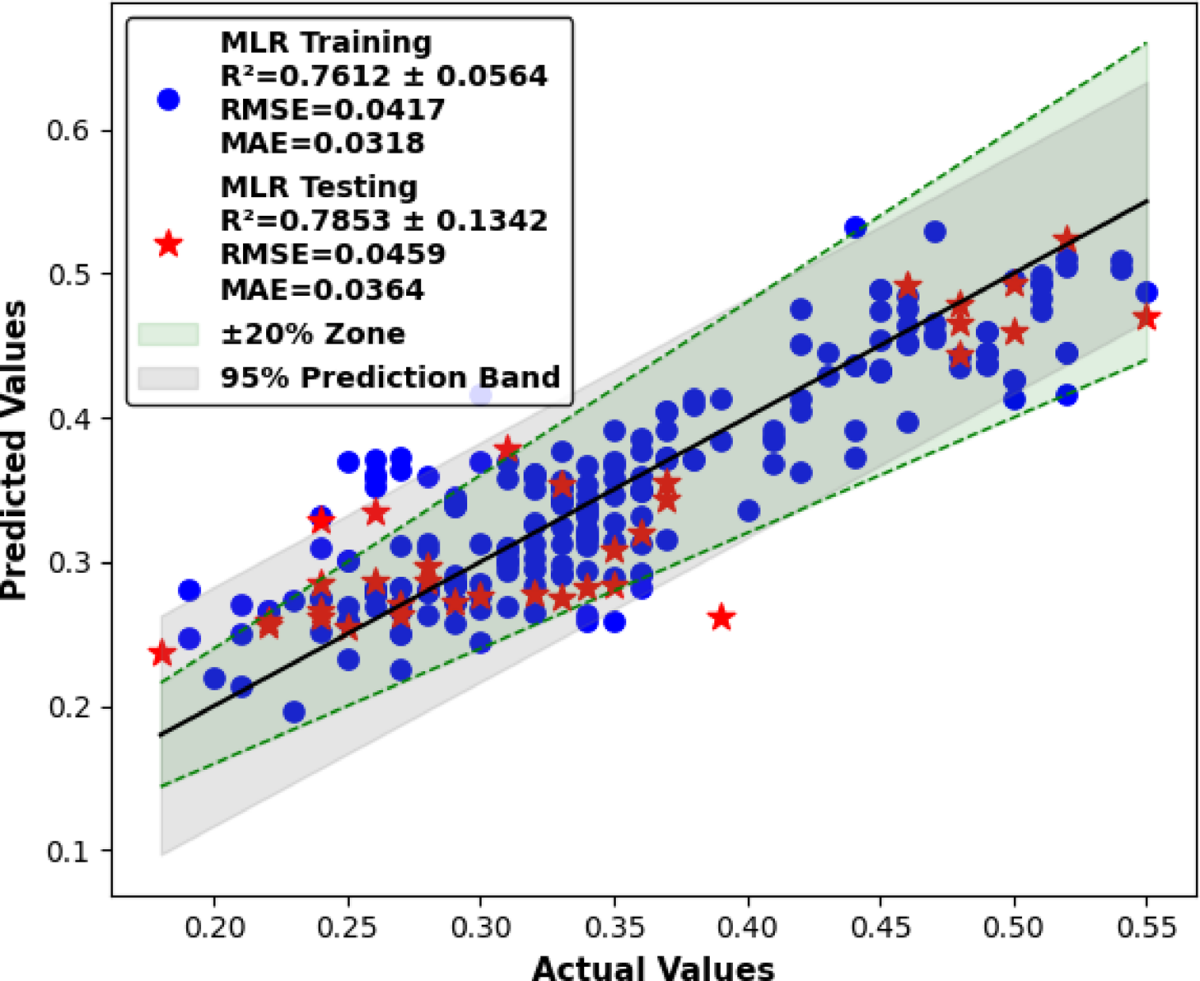
Predicted versus actual values for the MLR model.

The performance of the MLR model, proposed to develop the equation presented in Eq. 1, was extensively tested in both the training and testing sets, as shown in Fig. 4. It can be clearly observed that the MLR model provides results with excellent predictive power and produces a coefficient of determination (R^2^), reaching 0.7612 ± 0.0564 in the case of training and 0.7853 ± 0.1342 in the case of testing set. Furthermore, the small values of errors obtained by the MLR model for both training (RMS = 0.0417, MAE = 0.0318) and testing (RMS = 0.0459, MAE = 0.0364) phases, along with the fact that the majority of the data points lie within the ± 20 error zone and within the 95% prediction band marked as a shaded area, emphasize the high accuracy of the proposed empirical equation.

1 $$\begin{aligned} {\mathrm{y}} & = - 3.74083 \times 10^{ - 4} \cdot {\mathrm{x}}1 - 0.00401 \cdot {\mathrm{x}}2 - 0.00442 \cdot {\mathrm{x}}3 \\ & \quad - 0.00335 \cdot {\mathrm{x}}4 - 0.01674 \cdot {\mathrm{x}}5 - 0.2773 \cdot {\mathrm{x}}6 \\ & \quad + 0.76377 \cdot {\mathrm{x}}7 - 0.01739 \cdot {\mathrm{x}}8 - 0.19089 \cdot {\mathrm{x}}9 \\ & \quad + 0.21497 \cdot {\mathrm{x}}10 + 0.20306 \cdot {\mathrm{x}}11 + 0.00985 \cdot {\mathrm{x}}12 \\ \end{aligned}$$

where

- y = Shear strength of soil (kg/cm^2^)
- x1 = Depth of sample taken (m)
- x2 = Sand proportion (%)
- x3 = Loam proportion (%)
- x4 = Clay proportion (%)
- x5 = Moisture content (%)
- x6 = Wet density (g/cm^3^)
- x7 = Dry density (g/cm^3^)
- x8 = Void ratio
- x9 = Liquid limit (%)
- x10 = Plastic limit (%)
- x11 = Plastic index
- x12 = Liquidity index

#### Support vector machine model

**Fig. 5:**
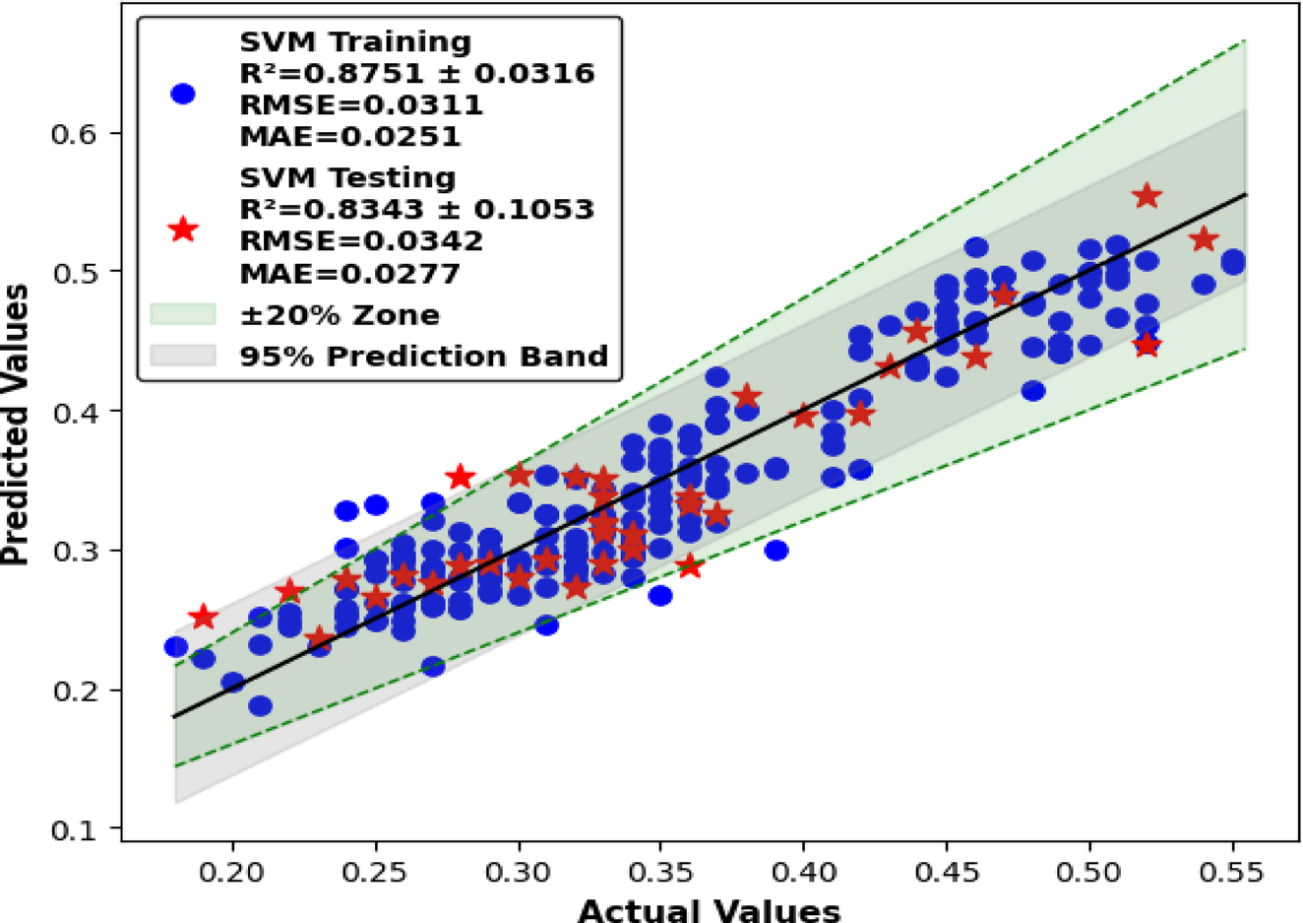
Predicted versus actual values for the SVM Model.

The predictive power and reliability of the SVM model have been analyzed in both training and testing data sets as shown in Fig. 5. The SVM model showed high levels of prediction ability and statistical reliability, giving a highly strong coefficient of determination (R^2^) value of 0.8751 ± 0.0316 in the training phase. Importantly, the high level of accuracy has been effectively retained in the testing phase as well, producing an R^2^ value of 0.8343 ± 0.1053. The insignificant difference between the two R^2^ values indicates high levels of generalizability in the model and the absence of any problem related to overfitting. The high precision level of the model is also confirmed by the low error values; the training phase resulted in RMSE and MAE values of 0.0311 and 0.0251, respectively, which are close to that of the testing data set (RMSE = 0.0342, MAE = 0.0277). From the graphical representation of the data, both data sets clustered very tightly on the ideal diagonal line.

#### Random forest model

**Fig. 6:**
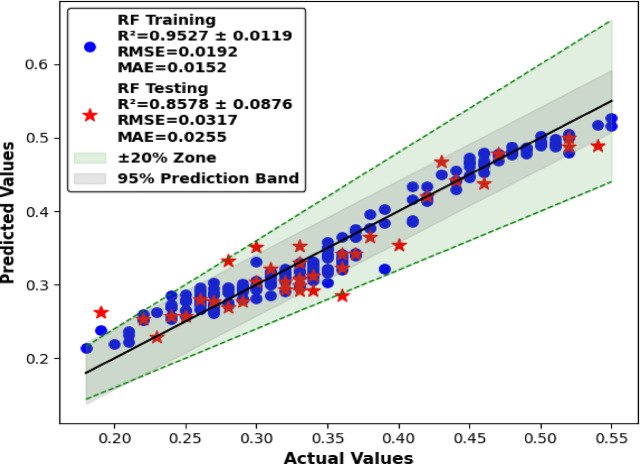
Predicted versus actual values for the RF Model.

Predictive capabilities and generalization capacity of the RF model were critically analyzed through analysis based on the training and test datasets, as illustrated in Fig. 6. The RF model showed excellent predictability with respect to the training dataset, where a very high coefficient of determination (R^2^) equal to 0.9527 ± 0.0119 was achieved, as well as extremely low values of error measures, like RMSE and MAE (RMSE = 0.0192 and MAE = 0.0152). During the analysis based on the testing dataset, which is independent of the training set, the RF model maintained its high level of predictability, since the calculated values of R^2^, RMSE, and MAE turned out to be equal to 0.8578 ± 0.0876, 0.0317, and 0.0255, respectively. As noted, before, although there is a reduction in predictive ability between training and testing datasets, which is a common characteristic of machine learning models, it is not significant due to the relatively small difference between these datasets. Additionally, visual inspection of Fig. 6 confirms high predictability of the proposed model as most predictions lie in the ± 20% error zone and 95% prediction interval.

#### Multi-expression programming model

**Fig. 7:**
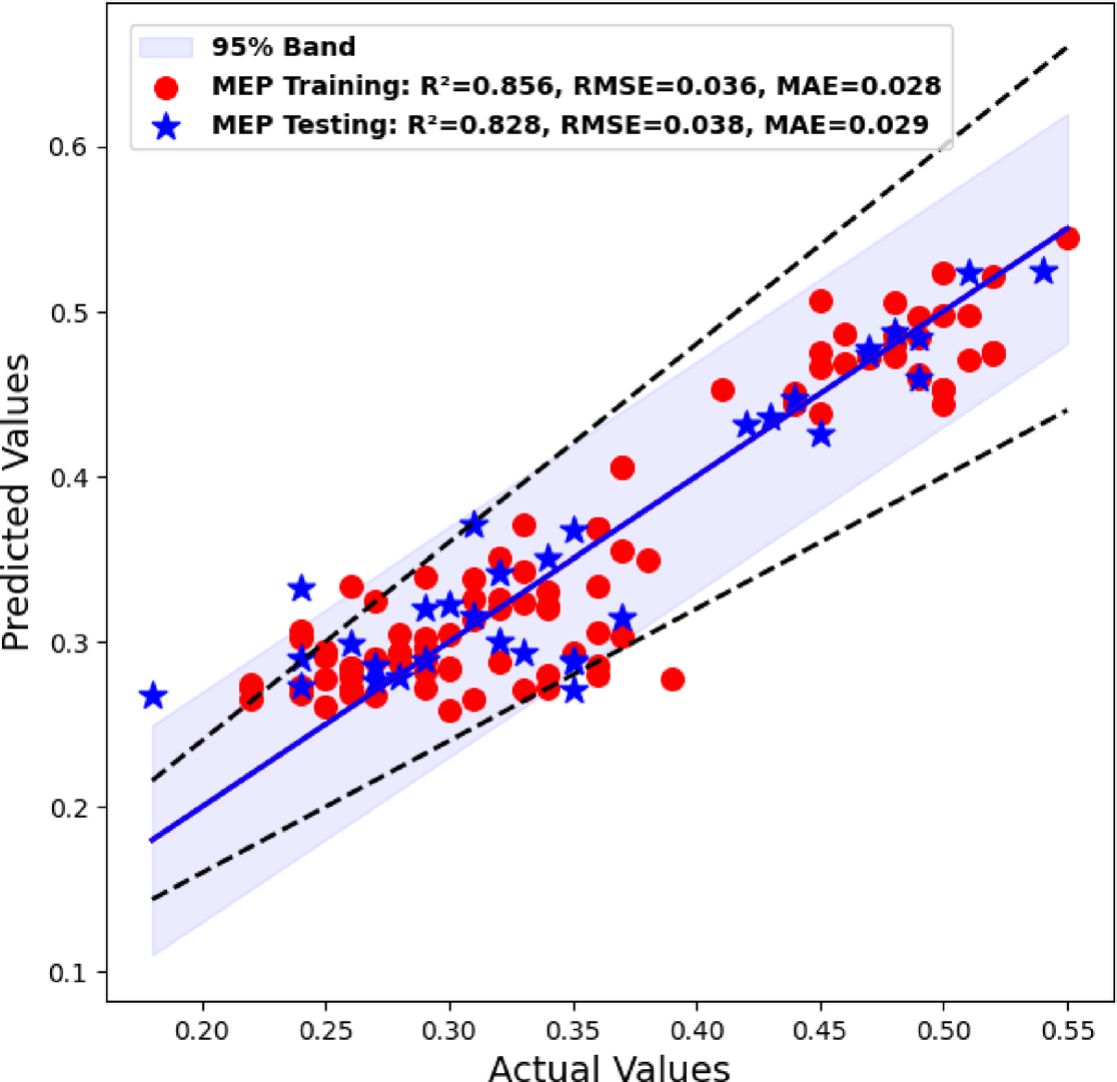
Predicted versus actual values for the MEP Model.

The MEP model predictive performance is a symbolic Regression as the actual and predicted values plot Fig. 7 confirms the fact that the model captures the underlying relationships in the available data, albeit slightly less precisely than the RF model. The scatter plot of the data points of the training and testing set is remarkably similar to the line of perfect agreement (1:1) with majority of the scatter being concentrated inside the 95% Confidence Band. The MEP model resulted in R^2^ = 0.856, RMSE = 0.036 and MAE = 0.028, which implies a good fit to the data in the case of the Training Set. Testing Set on the remaining data the model continued to give almost similar scores with R^2^ = 0.828, RMSE = 0.038 and MAE = 0.029, which shows the ability of the model to generalize well. MEP model is a good empirical model to predict Eq. 2 generated by MEP and can capture relationships that are complex in the data, and the model gives a more interpretable and explicit mathematical representation than the RF model does.

2 $$y\approx {x}_{11}^{{x}_{10}}+\frac{\frac{{x}_{6}}{{x}_{6}+{x}_{6}^{{x}_{1}}}+(\frac{A-B}{Cx11})/{E}^{{x}_{6}{x}_{11}}}{\sqrt{D}+\frac{A-B}{Cx11}}-\frac{(\frac{A-B}{Cx11})}{E/C}$$

where

1. $$A={x}_{1}+\sqrt{\left|{x}_{3}\right|}$$
2. $$B={x}_{4}^{{x}_{6}{x}_{11}^{2}}$$
3. $$C={x}_{6}^{\sqrt{\left|{x}_{3}\right|}}$$
4. $$D={\left({x}_{6}^{\sqrt{\left|{x}_{3}\right|}}\right)}^{\frac{{x}_{6}}{{x}_{1}}}={C}^{\frac{{x}_{6}}{{x}_{1}}}$$
5. $$E={\left({x}_{0}-{x}_{11}\right)}^{\frac{{x}_{0}}{\sqrt{D}}}$$
6. $$F=\frac{A-B}{C{x}_{11}}$$

- Y = Shear Strength (kg/cm^2^)
- x0 = Depth of the Sample (m)
- x1 = Sand Percentage
- x2 = Loam Percentage
- x3 = Clay Percentage
- x4 = Moisture Content (%)
- x5 = Wet Density (g/cm^3^)
- x6 = Dry Density (g/cm^3^)
- x7 = Void Ratio
- x8 = Liquid Limit (%)
- x9 = Plastic Limit (%)
- x10 = Plasticity Index
- x11 = Liquidity Index

### Residual analysis

**Fig. 8:**
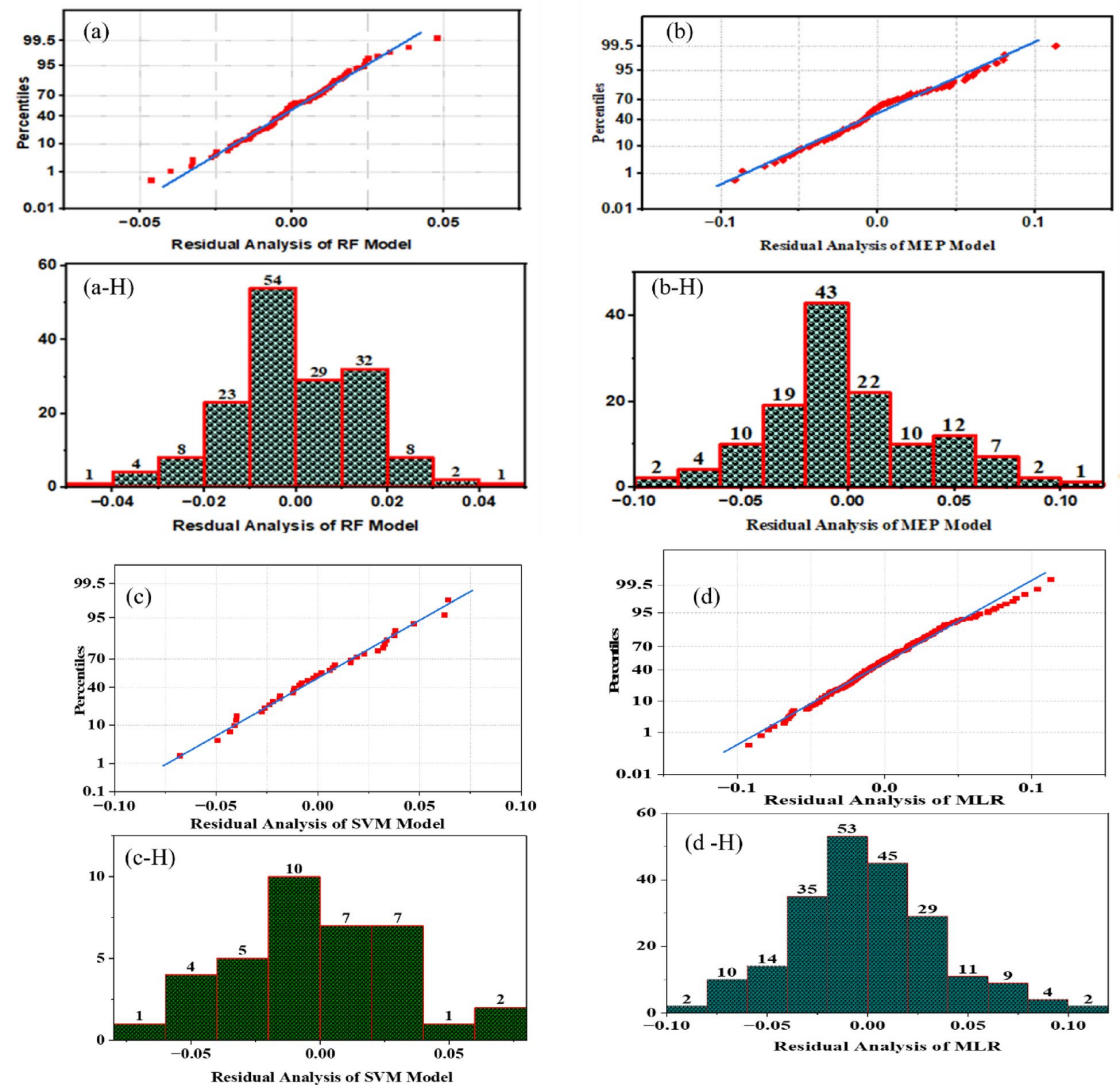
Residual analysis of ML models: (**a**, a-H) RF; (**b**, b-H) MEP; (**c**, c-H) SVM; (**d**, d-H) MLR. Upper panels show normal probability plots; lower panels (-H) show residual histograms.

Statistical rigor and accuracy of prediction of the ML model was also confirmed by Residual Analysis in Fig. 8^23^. The prediction accuracy, error distribution, and robustness of the generated machine learning models have been assessed using this residual analysis and normal probability plots for the RF, MEP, SVM, and MLR models. In all the four models, the residual data points tend to lie near the reference line in the normal probability plot, implying that the prediction errors roughly meet the requirement of normality assumption. Differences can be found among the four models regarding the spread of residuals. Residuals of the RF model have the minimum spread and lie closer to zero, representing a consistent model with low prediction errors. In addition, the SVM model also has a relatively small spread of residuals with symmetry, representing the good predictive ability and reliable stability of the model. As for the MEP model, the relatively large spread of the residual points shows the moderate predictive accuracy with more errors. In terms of the MLR model, the widest residual spread indicates its poor prediction ability.

The analysis results from the histogram support this observation where RF and SVM models show an exceptionally high concentration of residuals towards the zero-error region with minimal outliers, but MEP and MLR models show a much larger dispersion of residuals. In total, the best model would be identified as RF due to an extremely dense distribution of residuals, minimal spread of errors, highest similarity of predicted values to experimental ones, and high generalization ability of the model. SVM model was second in terms of performance, followed by MEP, and MLR was the worst among all the developed models regarding accuracy. It could be concluded that more sophisticated machine learning algorithms such as RF and SVM prove to work better on modeling the complex nonlinear dataset behavior than classic regression models.

### Taylor diagram comparison

**Fig. 9:**
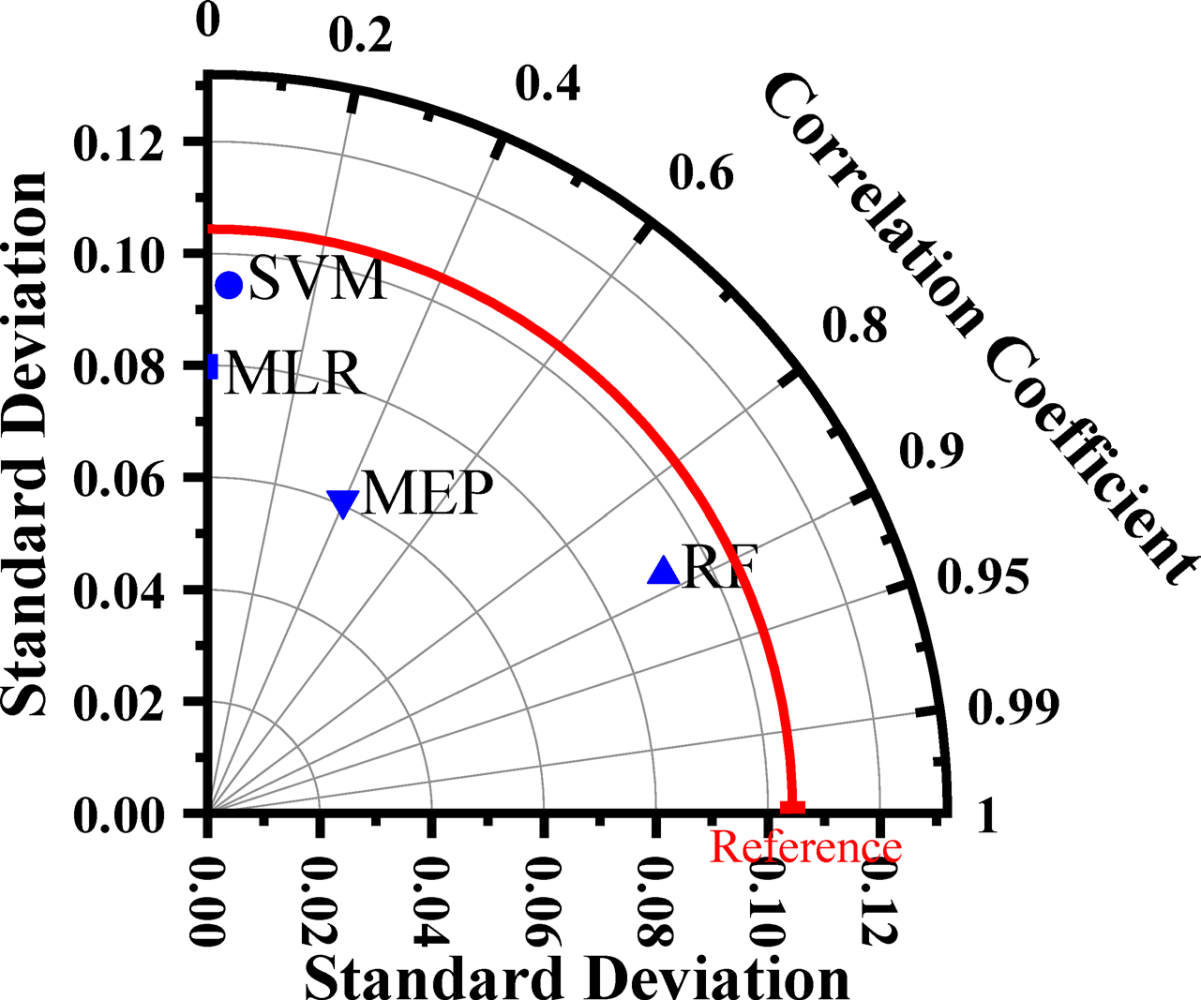
Taylor plot.

For a comprehensive analysis and comparison of the predictive accuracy of the constructed models (SVM, MLR, MEP, and RF), a Taylor diagram was employed shown in Fig. 9. In such a way, the graph allows depicting three different statistical measures simultaneously: correlation coefficient, standard deviation, and root-mean-square error (RMSE). Specifically, the distance from the center point to each model corresponds to the standard deviation of the constructed simulation, and the angle formed between each point and the vertical line is an indication of the correlation coefficient with the reference observations. Moreover, the distance between any point and the Reference point on the horizontal axis reflects the centered RMSE for a given model. As can be seen from the graph, the best results were achieved by the model based on Random Forest (RF), which lies closest to the Reference point. This model has the highest correlation coefficient and standard deviation close to the reference observation. On the contrary, the SVM, MLR, and MEP models are placed significantly further from the Reference point due to the poor linear correlation, higher RMSE, and non-uniform standard deviation.

### Error metrics summary

Table 3 shows the performance prediction of the developed models, showing both the training and training results of ML models. The RF model provided the best results in terms of the prediction accuracy (R^2^ = 0.9527 ± 0.0119 training, 0.8578 ± 0.0876 testing) and error minimization (RMSE = 0.0192 training, 0.0317 testing; MAE = 0.0152 training, 0.0255 testing) that proved its superiority among the considered models. The SVM model exhibited a strong capacity of nonlinear learning and generalization (R^2^ = 0.8751 ± 0.0316 training, 0.8343 ± 0.1053 testing) confirmed by reasonably low prediction errors. The MEP model provided decent and stable performance (R^2^ = 0.8560 ± 0.0400 training, 0.8280 ± 0.0920 testing) together with clear mathematical expressions that made it applicable in practice. Lastly, the MLR model showed lower predictive accuracy (R^2^ = 0.7612 ± 0.0564 training, 0.7853 ± 0.1342 testing) compared to other models because of poor generalization ability for nonlinear data, though it provided stable performance.

**Table 3:** Error Metrics.

Model	Training R^2^	Testing R^2^	Training RMSE	Testing RMSE	Training MAE	Testing MAE	Model Interpretation
MLR	0.7612 ± 0.0564	0.7853 ± 0.1342	0.0417	0.0459	0.0318	0.0364	Moderate predictive capability with stable generalization
SVM	0.8751 ± 0.0316	0.8343 ± 0.1053	0.0311	0.0342	0.0251	0.0277	Strong nonlinear learning and reliable prediction performance
RF	0.9527 ± 0.0119	0.8578 ± 0.0876	0.0192	0.0317	0.0152	0.0255	Highest predictive accuracy and strongest overall performance
MEP	0.8560 ± 0.0400	0.8280 ± 0.0920	0.036	0.038	0.028	0.029	Demonstrated good generalization capability and reliable prediction performance across independent testing data

### Comparison with past studies

The latest developments in ML have altered the role of empirical models in predicting soil shear strength to data-driven frameworks, which are more accurate as indicated in Table 4, resistant, and easy to comprehend. Other studies are based on RF and MEP models, which demonstrate that these strategies can provide complex nonlinear interaction of cohesion, plasticity index, and water content with consistency. RF offers higher generalization and interpretability with the analysis of feature importance, but MEP offers explicit symbolic representations, which promote a deeper physical comprehension. According to comparative analyses, it can be suggested that both RF and MEP provide high levels of predictive accuracy that frequently exceed R^2^ values of 0.90 and that both significantly lower the mean errors than traditional regression or ANN techniques. With these developments, the increased value of explainable ML in geotechnical engineering is highlighted and making more transparent, data-driven decision making in geotechnical designs and in stability assessment feasible.

**Table 4:** Summary of past study.

References	Mode	Soil Type	Key Results
^26^	Fuzzy Logic and ANN	Remolded soil	FL and ANN; R^2^ = 0.92 and 0.94 (Total dataset = 1306)
^27^	ANN	Reinforced Concrete	ANN achieved R^2^ = 0.97; generated interpretable equations (Total dataset = 115)
^28^	Gradient Boosting	Soil Shear Strength	GB achieved R^2^ = 0.99; key variables: water content, density (Total dataset = 249)
^29^	ANN, RF and XGB	undrained shear strength	Identified feature importance; cohesion and saturation dominant (Total dataset = 384)
^30^	Polynomial Regression Model	Fine Grained Soil	R^2^ = 0.85 (Total dataset = 50)
^31^	RF, Linear Regression, SVM	Slope Stability	R^2^ = 0.83, 0.86, 0.87 (Dataset = not mentioned)
^32^	slime mold algorithm (SMA	Shear Strength of Rock joint	R^2^ = 0.97 (Total dataset = 84)
^33^	RF, SVR ,SGB, Cubist model	Undrained Shear Strength	Cubist model R^2^ = 0.87 (Total dataset = 372)
^34^	NB, NBSH, NBAO	Clayey soils	R2 = 0.95, 0.98, 0.97 (Total dataset = 200)
^35^	AdaBoost, DT, CatBoost	Granular soils, Small-strain shear modulus	R^2^ = 0.959, 0.981, 0.994 (Total dataset = 653)

### SHapley additive ExPlanations (SHAP) analysis

**Fig. 10:**
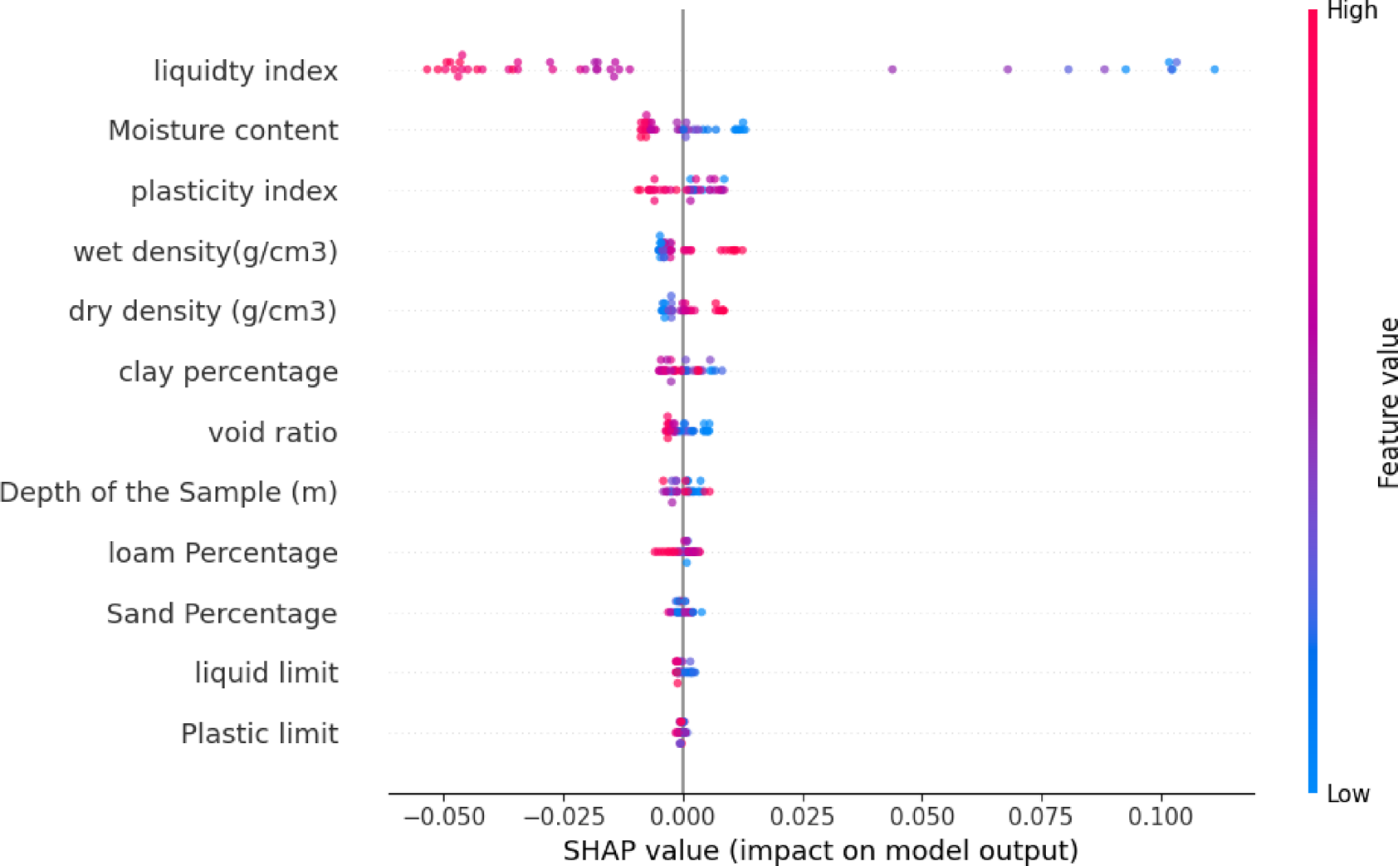
SHapley additive ExPlanationss (SHAP) analysis of RF model.

SHAP analysis was used to understand the Multifaceted black-box behavior of the RF model and estimate the effects of each input feature. Figure 10 shows the SHAP summary plot, the effect of the input variables on the model prediction is demonstrated. This plot not only provides the effects of all input variables but also allows us to rank them based on their importance. As shown, the most influential predictor in this model is the liquidity index, because this variable has the highest range of SHAP values. This suggests that changes in this predictor lead to larger changes in the predictions made by the model. When the liquidity index is lower (the blue points), the model output tends to be pushed towards larger positive SHAP values; however, when the liquidity index is high (the pink points), the model prediction decreases. Moisture content and plasticity index are the second most important predictors in this model. Other predictors such as wet density, dry density, percent clay, and void ratio also affect the output; however, their effects are rather minor. Liquid limit and plastic limit are considered the least important predictors in this model.

### Feature ablation and sensitivity analysis

**Fig. 11:**
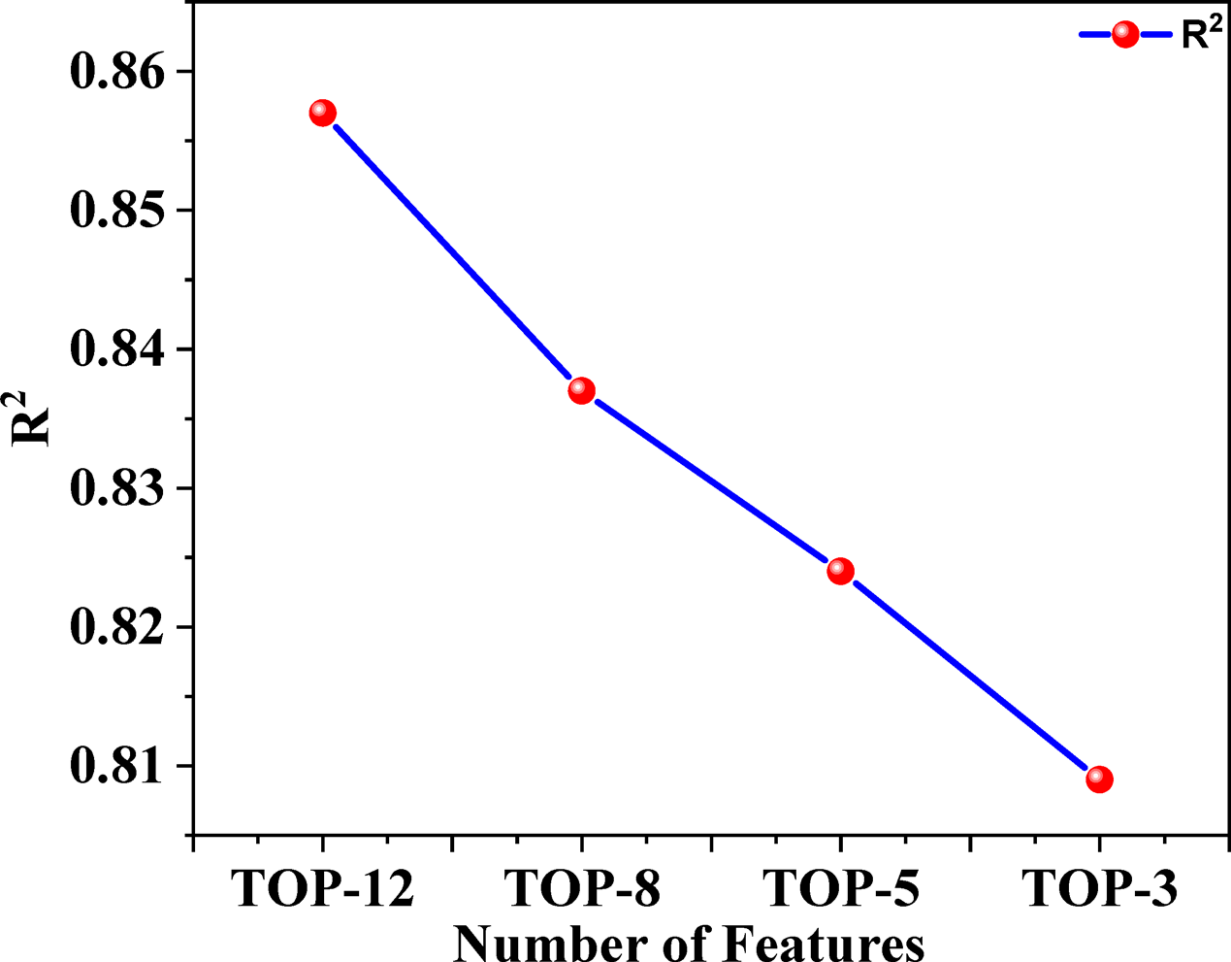
Performance degradation curve.

The practical robustness and applicability of the developed RF model were investigated using feature ablation analysis. In particular, the evaluation included studying the effect of feature deletion from the input vector on the prediction accuracy of different feature sets, where input features were selected based on the ranking of importance. Different feature vectors were used with decreasing numbers of features, from the initial set down to the top 8, top 5, and finally the top 3 most important features. Figure 11 shows the change in predictive performance depending on the subset feature size, reflecting the level of redundancy within the input variables. If the R^2^ of the model remains relatively stable with the decrease in the feature subset size, then some of the input parameters can be replaced by fewer features because of their high correlation. However, a sharp drop in the prediction accuracy indicates that secondary variables also make a significant contribution in describing the variability of the output response.

## Conclusion

In this study, the four proposed machine learning models including MLR, SVM, RF, and MEP were successfully developed and analyzed based on the geotechnical data of soil samples for the prediction of soil shear strength at the Le Trong Tan Geleximco project site, Hanoi, Vietnam. Several important findings are summarized below:

1. The RF model demonstrated superior predictive performance among all four models, achieving training R^2^ = 0.9527 and testing R^2^ = 0.8578 with the lowest error values (testing RMSE = 0.0317, MAE = 0.0255). Its ensemble-based structure effectively captured the nonlinear and complex interactions among soil properties, making it the most reliable model for soil shear strength prediction.
2. The SVM model offered strong generalization capability with a testing R^2^ = 0.8343 and low error metrics (RMSE = 0.0342, MAE = 0.0277), confirming its suitability for nonlinear geotechnical regression problems when an interpretable equation is not required.
3. The MEP model achieved competitive accuracy (testing R^2^ = 0.8280, RMSE = 0.038, MAE = 0.029) while providing an explicit symbolic mathematical equation governing shear strength prediction. This unique attribute makes MEP particularly valuable in engineering practice where transparent, human-readable relationships between soil properties are desired for design validation and regulatory compliance.
4. The MLR model, while providing a useful baseline and interpretable linear equation, yielded the lowest predictive accuracy (testing R^2^ = 0.7853), confirming the inherently nonlinear nature of soil shear strength and the inadequacy of linear approaches for such complex geotechnical problems.
5. Residual analysis and Taylor diagram comparisons consistently reinforced the ranking of model performance: RF > SVM > MEP > MLR, with RF and SVM residuals exhibiting the smallest spread and highest symmetry about zero, indicative of reliable and unbiased predictions.
6. SHAP (SHapley Additive exPlanations) analysis of the RF model identified the liquidity index as the most dominant predictor of soil shear strength, followed by moisture content and plasticity index. Features such as wet density, dry density, and clay percentage exhibited comparatively lower influence. These findings are physically consistent with fundamental soil mechanics theory and provide actionable insights for site investigation planning and parameter prioritization.
7. The strict data preprocessing pipeline, including outlier removal and Min-Max normalization applied to 202 curated samples, contributed significantly to the robustness and generalizability of all developed models.
8. This research establishes a foundation for integrating explainable ML frameworks into routine geotechnical practice, combining predictive power with physical interpretability. Future studies may benefit from expanding the dataset across diverse geological settings, incorporating additional parameters such as specific gravity and stress history, and exploring deep learning architectures or hybrid optimization techniques to further enhance predictive performance.

Overall, this study underscores the significant potential of machine learning-based frameworks, especially when augmented with explainability tools, in advancing data-driven geotechnical engineering. The integration of RF’s predictive power with SHAP-based interpretability represents a step toward transparent, reliable, and efficient geotechnical design practices.

## Data Availability

Data will be made available upon request.
